# Source Attribution of Human Campylobacteriosis Using Whole-Genome Sequencing Data and Network Analysis

**DOI:** 10.3390/pathogens11060645

**Published:** 2022-06-03

**Authors:** Lynda Wainaina, Alessandra Merlotti, Daniel Remondini, Clementine Henri, Tine Hald, Patrick Murigu Kamau Njage

**Affiliations:** 1 Department of Mathematics, University of Padova, 35121 Padova, Italy; lyndanduta.wainaina@studenti.unipd.it; 2 Department of Physics and Astronomy, University of Bologna, 40126 Bologna, Italy; alessandra.merlotti2@unibo.it (A.M.); daniel.remondini@unibo.it (D.R.); 3 Research Group for Genomic Epidemiology, National Food Institute, Technical University of Denmark, 2800 Kgs. Lyngby, Denmark; clehen@food.dtu.dk; 4 Research Group for Foodborne Pathogens and Epidemiology, National Food Institute, Technical University of Denmark, 2800 Kgs. Lyngby, Denmark; tiha@food.dtu.dk

**Keywords:** source attribution, *Campylobacter*, campylobacteriosis, network analysis, whole-genome sequencing, coherence source clustering

## Abstract

*Campylobacter* spp. are a leading and increasing cause of gastrointestinal infections worldwide. Source attribution, which apportions human infection cases to different animal species and food reservoirs, has been instrumental in control- and evidence-based intervention efforts. The rapid increase in whole-genome sequencing data provides an opportunity for higher-resolution source attribution models. Important challenges, including the high dimension and complex structure of WGS data, have inspired concerted research efforts to develop new models. We propose network analysis models as an accurate, high-resolution source attribution approach for the sources of human campylobacteriosis. A weighted network analysis approach was used in this study for source attribution comparing different WGS data inputs. The compared model inputs consisted of cgMLST and wgMLST distance matrices from 717 human and 717 animal isolates from cattle, chickens, dogs, ducks, pigs and turkeys. SNP distance matrices from 720 human and 720 animal isolates were also used. The data were collected from 2015 to 2017 in Denmark, with the animal sources consisting of domestic and imports from 7 European countries. Clusters consisted of network nodes representing respective genomes and links representing distances between genomes. Based on the results, animal sources were the main driving factor for cluster formation, followed by type of species and sampling year. The coherence source clustering (CSC) values based on animal sources were 78%, 81% and 78% for cgMLST, wgMLST and SNP, respectively. The CSC values based on *Campylobacter* species were 78%, 79% and 69% for cgMLST, wgMLST and SNP, respectively. Including human isolates in the network resulted in 88%, 77% and 88% of the total human isolates being clustered with the different animal sources for cgMLST, wgMLST and SNP, respectively. Between 12% and 23% of human isolates were not attributed to any animal source. Most of the human genomes were attributed to chickens from Denmark, with an average attribution percentage of 52.8%, 52.2% and 51.2% for cgMLST, wgMLST and SNP distance matrices respectively, while ducks from Denmark showed the least attribution of 0% for all three distance matrices. The best-performing model was the one using wgMLST distance matrix as input data, which had a CSC value of 81%. Results from our study show that the weighted network-based approach for source attribution is reliable and can be used as an alternative method for source attribution considering the high performance of the model. The model is also robust across the different *Campylobacter* species, animal sources and WGS data types used as input.

## 1. Introduction

Human campylobacteriosis is among the most common zoonotic diseases, caused mainly by the bacteria *Campylobacter jejuni* and *Campylobacter coli*. Campylobacteriosis continues to be a major problem worldwide, including Denmark, which has seen the number of cases rising from 4547 in 2018 to 5389 in 2019. The increase in cases in Denmark was attributed to a large outbreak in chicken meat [1,2]. The main sources of human infection have been attributed to contaminated meat, poultry, water, milk and contact with farm animals [3]. Considering that many human campylobacteriosis cases have been attributed to various animal sources, there is a need to determine the relative contribution of the different exposures from animals to the total number of human cases [4].

Source attribution, which apportions human infection cases to different animal species and food reservoirs, has been instrumental in control- and evidence-based intervention efforts. Several methods for source attribution are available, including the microbial subtyping approach and comparative exposure assessment approach. Microbial subtyping involves characterizing isolates of specific pathogens by phenotypic and genotypic subtyping methods. The principle for this approach involves comparing isolates from different food and animal sources with those from humans. The comparative exposure assessment approach, on the other hand, determines the relative importance of the known transmission routes by estimating the human exposure to the pathogen through each route [5].

The microbial subtyping attribution approach has been proven to be a valuable source attribution method as it assumes that the distribution of subtypes in the collection of microbial isolates for each source used in the attribution exercise is similar to the true distribution of subtypes in each source. There are two main types of microbial subtyping attribution models: the frequency-matched attribution model, which compares human strain types in the sources and population genetic models based on modeling the organism’s evolutionary history [6,7]. Previous studies have reported several applications of the microbial subtyping approach including source attribution of human salmonellosis which was developed in Denmark [4,5,8]. The use of multilocus sequence typing (MLST) is another common example of the microbial subtyping approach, which has been used to identify lineages in bacterial populations by indexing the variation present in seven housekeeping genes located in various parts of the chromosome [9]. MLST data have been previously utilized to attribute the sources of human *C. jejuni* infections in New Zealand, as well as *Salmonella* in Denmark, using the Danish Salmonella source account model and the ClonalFrame algorithm [4,9].

Whole-genome sequencing (WGS) has been proven to be the most informative approach for the characterization of bacterial isolates and has been used to analyze multiple bacterial outbreaks, such as tuberculosis, listeriosis and salmonellosis, among others [10,11,12]. WGS data sets have become increasingly available. However, one of the limitations of WGS data is the complexity in data analysis due to variable gene content and difficulties interpreting obtained results [13]. Despite this, many studies have suggested approaches to overcome the limited discriminatory power of MLST by exploiting WGS data. These approaches can be grouped into methods based on the core genome or whole genome multilocus, termed gene-by-gene approaches and single-nucleotide polymorphism (SNP) detection, which segregate by host [14]. The gene-by-gene approaches assess the diversity of isolates based on alleles found for all wgMLST or cgMLST genes of the species of interest [15] while SNP-based methods distinguish isolates based on SNPs present in the entire genome, including the intergenic regions, potentially offering a higher resolution [16,17].

Different approaches have been used for source attribution using WGS data sets, including machine learning which has previously been applied in source attribution for *Salmonella enterica*, *Escherichia coli* and *C. jejuni* [13,18,19,20] and to predict the severity or outcome of microbial infections [21,22,23,24,25]. The machine learning approach involves training different algorithms and obtaining the best-performing model while obtaining the attribution probabilities of human isolates to different sources. Network analysis, on the other hand, has recently been demonstrated as an accurate approach for the source attribution of human salmonellosis [4]. Network analysis is based on weighted networks theory, where pairwise distance matrices from source attribution can be visualized as fully connected networks. Nodes in this theory correspond to *Campylobacter* isolates and links correspond to genetic distances. Weaker links imply greater genetic distance between isolates. Network analysis is useful in extracting network communities corresponding to different animal sources, where network communities correspond to groups of vertices with a higher probability of being connected to each other than other members of that group [26].

The probability of a human isolate to be associated with an animal source is computed as the function of the number of links that the human isolate has with other animal isolates. A specific animal source to which human genomes of *Campylobacter* are attributed can also be extracted from the network analysis. Using the network approach, we can identify which structural features of a data set play a fundamental role in determining the internal coherence of clusters [4], such as animal sources, species type and year of origin, etc. We demonstrated the potential of weighted networks for source attribution of human campylobacteriosis using whole-genome sequencing data. We compared the effect of different types of WGS data inputs namely cgMLST, wgMLST and SNP on the accuracy of the weighted network-based source attribution models.

## 2. Materials and Methods

### 2.1. Data Set

The data used in this study were collected between May 2015 and March 2017 from *Campylobacter* monitoring projects in Denmark. The data set was composed of 283 *C. jejuni* isolates and 434 other unknown *Campylobacter* species isolated from chickens, cattle, pigs, dogs, ducks and turkeys. The test material used were intestinal content (swabs, stools or appendices) and meat from various products collected either in a slaughterhouse or in the retail trade, originating from Danish or foreign production as well as the production environment. The *Campylobacter* isolates’ metadata were obtained from the Danish Food and Veterinary Administration (foedevarestyrelsen) and the sequenced genomes were extracted from the Center for Genetic Epidemiology (Food Institute Section of Computerome). The following information from the databases was used: sample ID, year of collection, country of origin and source (host of the *Campylobacter* isolate).

**Figure 1:**
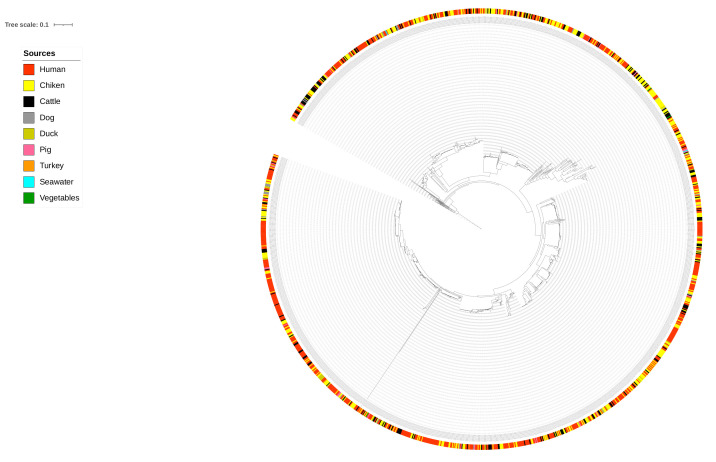
Phylogenetic tree from SNP distance matrix.

The human cases data set consisted of isolates received from Statens Serum Institute’s surveillance from January 2015 to December 2017. Isolates from humans with known travel history were not included in the data set. Data cleaning was performed to remove duplicates and isolates with incomplete metadata. The input data set for the network analysis consisted of cgMLST and wgMLST distance matrices from 717 food and 717 human isolates and SNPs from 720 food and 720 human isolates. The SNP distance matrix contained more isolates than cgMLST and wgMLST due to more matching isolate identification codes between the SNP data and the source metadata. The population structure was obtained from the phylogenetic analysis, as shown in Figure 1. This indicated that human isolates intermixed with other food and animal isolates, indicating that human *Campylobacter* strains were more likely to have originated from the sources described. Furthermore, sources were also genetically well distributed within the tree. Seawater and vegetables were omitted from the final input data used for network analysis since they were few and would have resulted in unreliable source attribution of human *Campylobacter* cases. All isolates used can be found under the bioproject number set up by the Statens Serum Institute, PRJEB31119 [27].

### 2.2. Bioinformatics Analysis

#### 2.2.1. Assemblies

The raw reads were de novo assembled. The procedure was done using the Food QC & Assembly pipeline, which includes assembler SPAdes version 3.9 [28]. The quality of the assembly was assessed using the number of contigs, N50, and the total size of the assembly. Assemblies were scaffold assemblies; genome assemblies with less than 500 contigs were kept in the data set. Eventually, the total size of the assembly was checked to match the expected size for a *C. jejuni* genome, which is between 1.6 to 1.7 million base pairs (Mbp).

#### 2.2.2. cgMLST and wgMLST

Core genome multilocus sequence typing (cgMLST) compares allelic profiles of several loci. CgMLST includes the core genome of *Campylobacter* and contains 1343 genes, as defined by Cody et al. in 2017 [29]. We performed core genome multilocus sequence typing (cgMLST) using the scheme developed by Cody *et al.* [29] available from the Center for Genomic Epidemiology pipeline [30]. Similarly, the wgMLST scheme used in this work includes 1643 genes from the re-annotation of the genome sequence of reference *C. jejuni* genome NCTC 11,168 [31].

#### 2.2.3. SNP

The SNP matrix was built using the CSI phylogeny pipeline accessible from the Center for Genomic Epidemiology [30,32]. The paired-end reads were mapped to the reference genomes using Burrows-Wheeler Aligner (BWA) [33]. The SNP analysis was performed using the reference genome: *C. jejuni* subsp. *jejuni* NCTC 11168 = ATCC 700819 (accession NC 002163.1). SNPs were determined using mpileup commands from SAMTools version 0.1.18 [34,35]. The SNPs were filtered according to five parameters: a minimum distance of 10 bps between each SNP, a minimum of 10x depth and 10 percent of the breadth coverage, the mapping quality was above 30, the SNP quality was higher than 20 and all indels were excluded [28,29,32,33,34]. For each genome, SNPs were concatenated to a single alignment corresponding to the positions of the reference genome. ItoL version 6 was used for the visualization of the phylogenetic tree, where the number of SNPs between isolates is equivalent to the distance in the tree [36].

All bioinformatic analysis were performed using Danish National Supercomputer for Life Sciences, Computerome 2.0, a local server for a Linux-based command-line system [37].

### 2.3. Network Analysis

The weighted network approach was used in this study, where the pairwise distance matrix was represented as a network with nodes corresponding to human *Campylobacter* isolates and links as a function of the pairwise distance. This pairwise distance was calculated as the number of different MLST alleles or SNPs between two isolated sequences. The assumption is that genomes coming from the same source show smaller distances. A fully connected weighted network, with weight calculated as 1/distance assigned to each link, was built in MATLAB [38]. A threshold was applied such that, in the resulting binary network, nodes were connected by an edge if the weight was greater than the threshold. The threshold was applied to remove weaker links with larger genetic distances, and it was chosen to maximize the internal coherence of clusters and minimize the number of isolated nodes. In the resulting binarized network, nodes were linked with an edge only if their weight was greater than the threshold value and clusters identified using the thresholding procedure [4].

The best threshold values were obtained using a 70/30 cross-validation procedure on the animal source data and were chosen in order to maximize the internal coherence of clusters (CSC, Equation (1) [4]) and minimize the number of isolated nodes. The 70/30 cross-validation procedure involved randomly selecting a network training set consisting of 70% of animal origin samples and using this set to obtain a best threshold value. This threshold value was applied to the network constructed using the test set composed of the remaining 30% of the animal samples for the calculation of the CSC as shown in Equation (1). This procedure was repeated 100 times and the most frequent threshold value was selected as the best overall threshold for further use in source clustering. The best threshold was then applied to the full pairwise distance matrix consisting of both animal and human isolates such that the human sources could be attributed to specific animal sources [4]. The best threshold was used to maximize the score function on distance matrices, as shown in Equation (2) [4]. The graphical visualizations of the network were obtained using the MATLAB ‘Plot’ function with the force-directed graph layout [39].

(1) $$CSC=\frac{\sum_{i=1}^{N_{c}}TP_{i}}{\sum_{i=1}^{N_{c}}T_{i}}100$$

(2) $$Score=\left(1-\frac{N_{ISO}}{N_{TOT}}\right)CSC$$

$N_{TOT}$ is the total number of nodes in the network, while $N_{ISO}$ is the number of isolated nodes that do not have any links to other nodes. *CSC* is the coherent source clustering, which measures the algorithm’s clustering performance, where $TP_{i}$ is the number of true positives in the $i^{th}$ cluster (majority of isolates from the same source in the same cluster) and $T_{i}$ is the total number of nodes inside the $i^{th}$ cluster [4].

## 3. Results and Discussion

**Figure 2:**
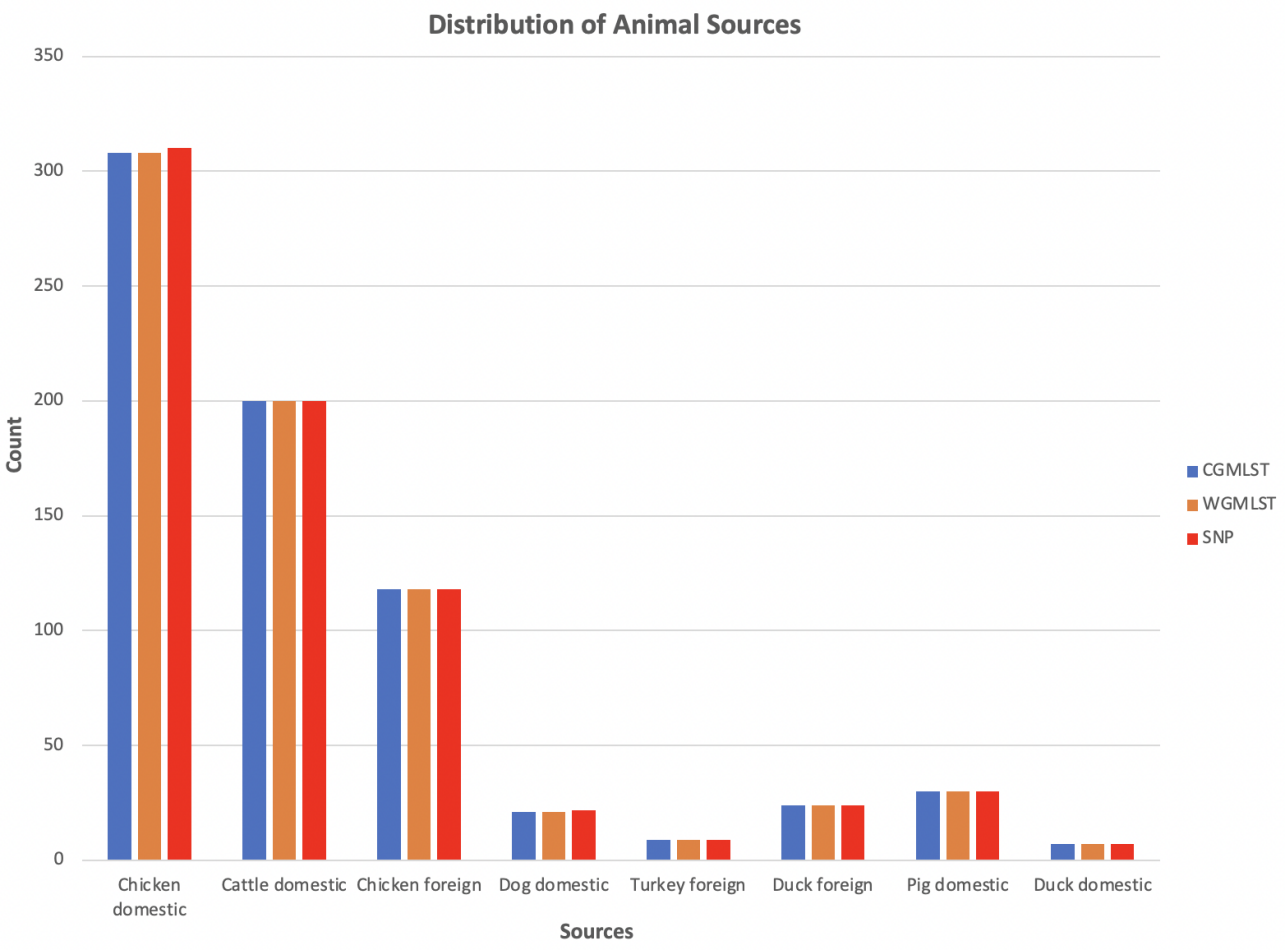
Distribution of animal sources used as input data for network analysis.

**Figure 3:**
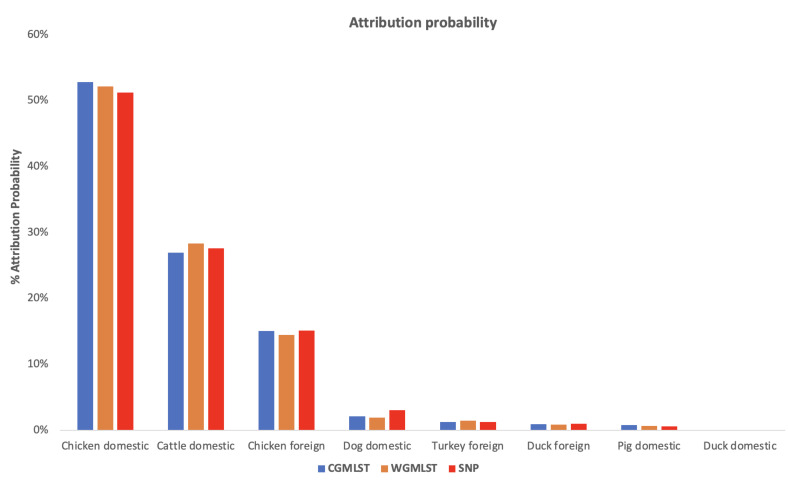
Mean probability (in percentage) of a human isolate to be attributed to a source, calculated for each of the considered pairwise distance matrices (cgMLST, wgMLST and SNP).

We compared results obtained using cgMLST, wgMLST and SNP distance matrices from the network analysis. Figure 2 shows the distribution of input data which corresponded to Figure 3 indicating the mean percentage attribution probability for the three distance matrices. We observed that chickens from Denmark were the main sources of human campylobacteriosis cases, with a percentage of attribution of 52.84%, 52.17% and 51.22% for cgMLST, wgMLST and SNP, respectively, while ducks from Denmark were the least probable source of infection. These results are in harmony with previous reports showing chicken meat as the main source of campylobacteriosis in Denmark [3]. The best threshold values obtained from the cross-validation were 0.1141, 0.0105 and 1715 for cgMLST, wgMLST and SNP distance matrices, respectively (Table 1). These values were used to maximize the score function from 100 runs of cross-validation.

**Figure 4:**
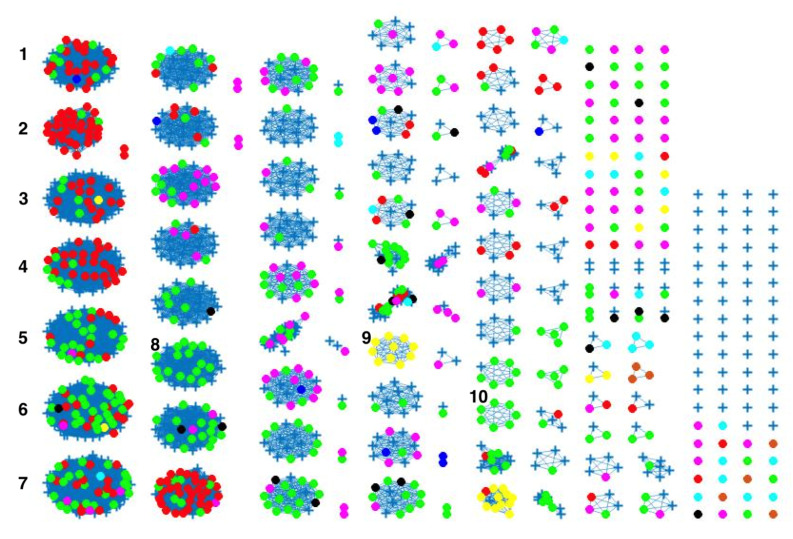
Source clustering results (force-directed graph drawing algorithm) obtained using cgMLST distance matrix as model input. Nodes represent different animal isolates. Cluster number 1–7 (misclassification of isolates within the cluster), 8–10 (correct classification of isolates within the cluster). Legend: red—cattle from Denmark; green—chickens from Denmark; magenta—chickens from foreign countries; black—dogs from Denmark; dark blue—turkeys from foreign countries; yellow—pigs from Denmark; cyan—ducks from foreign countries; light brown—ducks from Denmark; blue crosses—Human isolates. Foreign (Germany, Netherlands, Italy, France, Poland, UK, Hungary).

**Figure 5:**
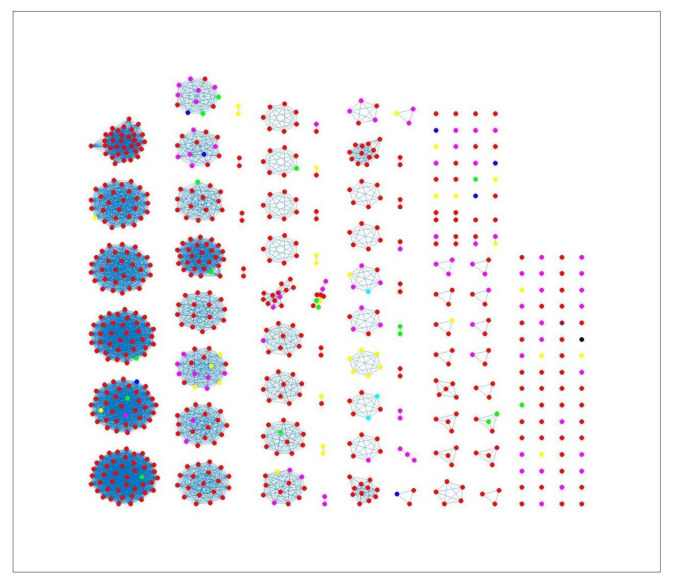
Clustering results (force-directed graph drawing algorithm) obtained using cgMLST distance matrix as model input. Nodes represent country of origin for different animal isolates. Legend: red—Denmark; yellow—Poland; magenta—France; green—Germany; blue—Netherlands; cyan—Italy; black—UK; brown—Hungary.

**Figure 6:**
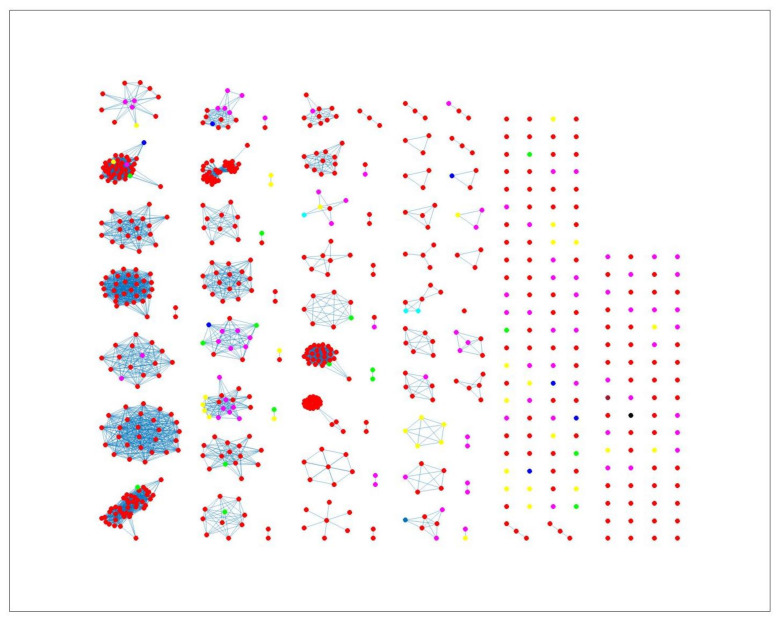
Clustering results (force-directed graph drawing algorithm) obtained using wgMLST distance matrix as model input. Nodes represent country of origin for different animal isolates. Legend: red—Denmark; yellow—Poland; magenta—France; green—Germany; blue—Netherlands; cyan—Italy; black—UK; brown—Hungary.

**Figure 7:**
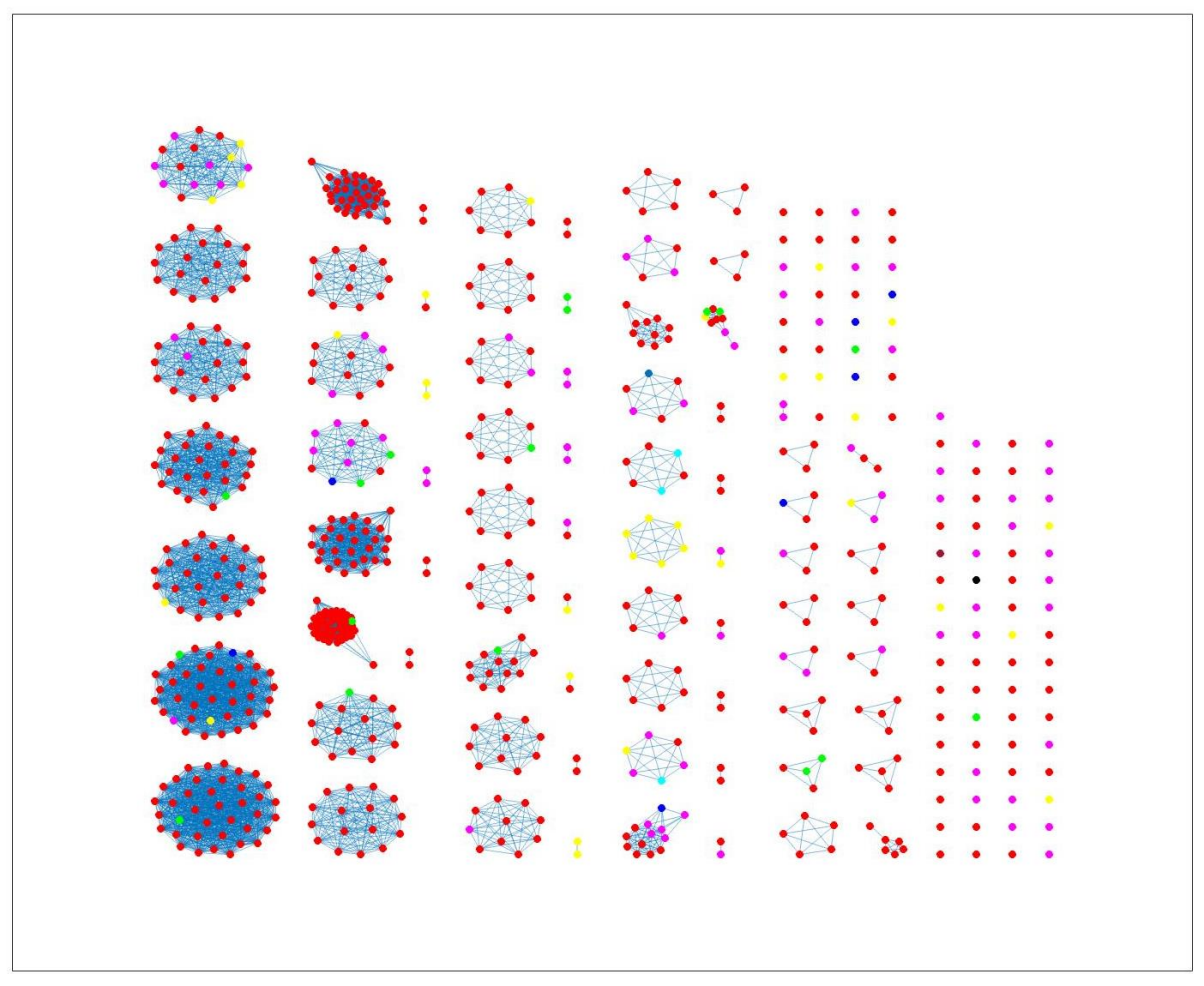
Clustering results (force-directed graph drawing algorithm) obtained using SNP distance matrix as model input. Nodes represent country of origin for different animal isolates. Legend: red—Denmark; yellow—Poland; magenta—France; green—Germany; blue—Netherlands; cyan—Italy; black—UK; brown—Hungary.

The network-based method achieved 78%, 81% and 78% coherent source clustering for cgMLST, wgMLST and SNP distance matrices, respectively (Table 1). The results indicated that animal sources were the main factors driving the clustering, followed by type of *Campylobacter* species and finally year of origin (Table 2). Table 3 shows results from adding human isolates to the network, where 88%, 77% and 88% were clustered within the existing animal network for cgMLST, wgMLST and SNP, respectively, while the remaining isolates were not to linked to *Campylobacter* genomes from any of the animal sources. The algorithm performed reasonably well in source attribution. However, some isolates were wrongly classified. For example, 43 chicken isolates were classified as cattle isolates Table 4. This misclassification is also apparent in Figure 4, where different sources in the main clusters 1, 4 and 5 from network analysis using cgMLST distance matrix as input data cannot be clearly distinguished. A consideration of the country of origin of animal sources showed that regionality affects cluster formation, as seen in Figure 5, Figure 6 and Figure 7, where most isolates are clustered according to origin.

**Figure 8:**
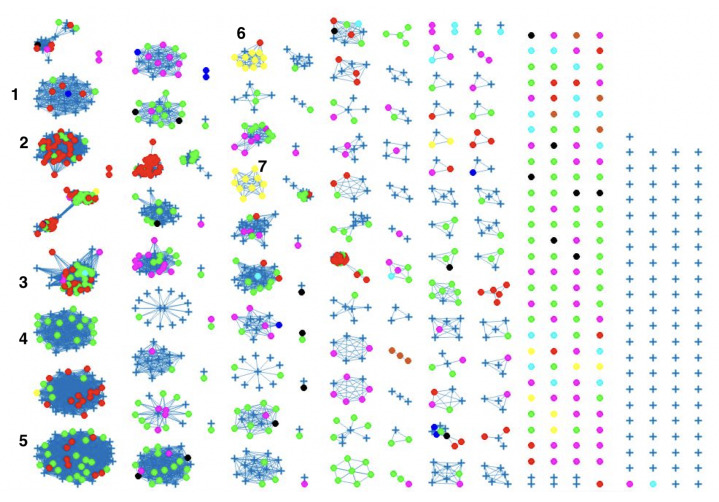
Source clustering results (force-directed graph drawing algorithm) obtained using wgMLST distance matrix as model input. Nodes represent different animal isolates. Cluster number 1–3, 5–6 (misclassification of isolates within the cluster) 4 and 7 (correct classification of isolates within the cluster). Legend: red—cattle from Denmark; green—chickens from Denmark; magenta—chickens from foreign countries; black—dogs from Denmark; dark blue—turkeys from foreign countries; yellow—pigs from Denmark; cyan—ducks from foreign countries; light brown—ducks from Denmark; blue crosses—Human isolates.

**Figure 9:**
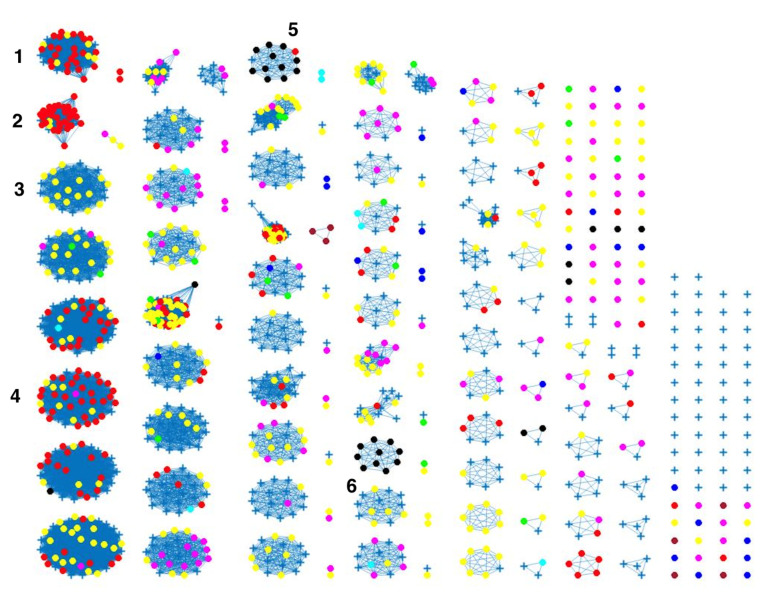
Source clustering results (force-directed graph drawing algorithm) obtained using SNP distance matrix as model input. Nodes represent different animal isolates. Cluster number 1, 2, 4, 5 (misclassification of isolates within the cluster), 3 and 6 (correct classification of isolates within the cluster). Legend: red—cattle from Denmark; yellow—chickens from Denmark; magenta—chickens from foreign countries; green—dogs from Denmark; cyan—turkeys from foreign countries; black—pigs from Denmark; blue—ducks from foreign countries; light brown—ducks from Denmark; blue crosses—Human isolates.

We noted that despite the class imbalance in the input data (Figure 2), the less abundant sources, such as dogs from Denmark, still had 100% human isolates linked to the sources, as shown in Figure 4, Figure 8 and Figure 9. This is an indication that sample imbalance does not affect source attribution using the network analysis method [4] and that the most consumed animal sources are most likely to cause the majority of *Campylobacter* infections. Class imbalances in other models lead to important patterns in the predictors being associated with the larger classes which results in less predictions for classes with less samples [40]. The best-performing model was the one with the wgMLST distance matrix as the input data, which had a CSC value of 81%. We calculated the confusion matrix for the cgMLST, wgMLST and SNP distance matrices’ clustering results (Table 4, Table 5 and Table 6). The weighted network analysis approach provided quite good results considering the model performance in comparison to other source attribution models [13,18,19,20] and microbial infection severity and outcome prediction models such as machine learning [21,22,23,24,25].

The F1 scores calculated from the confusion matrices above were: 75.96%, 79.94% and 74.93% for cgMLST, wgMLST and SNP, respectively. The best-performing model from the F1 score was based on wgMLST distance matrix as input data which is also in agreement to the model’s high CSC value of 81%.

Similar clustering results were observed from the network analysis approach as observed above. Figure 8 indicates some confusion in distinguishing between different sources. For example, in cluster 2, there is no proper separation between chickens and cattle from Denmark, which is also observed in Figure 9 (clusters 1, 2, 4). However, a high proportion of the food sources where less isolates were available such as pigs, were attributed to human cases as observed in cluster 6 in Figure 8. The results from the wgMLST distance matrix input data show that the network-based algorithm performs best in clustering considering the high CSC value of 81%. The results in Figure 6 and Figure 7 show that the region of origin of the animal sources has an influence on cluster formation. Considering that most of the animal isolates are from Denmark, the main clusters are dominated by Danish isolates, with some clusters consisting of less abundant isolates such as imports from Poland, as observed in Figure 5, Figure 6 and Figure 7.

**Figure 10:**
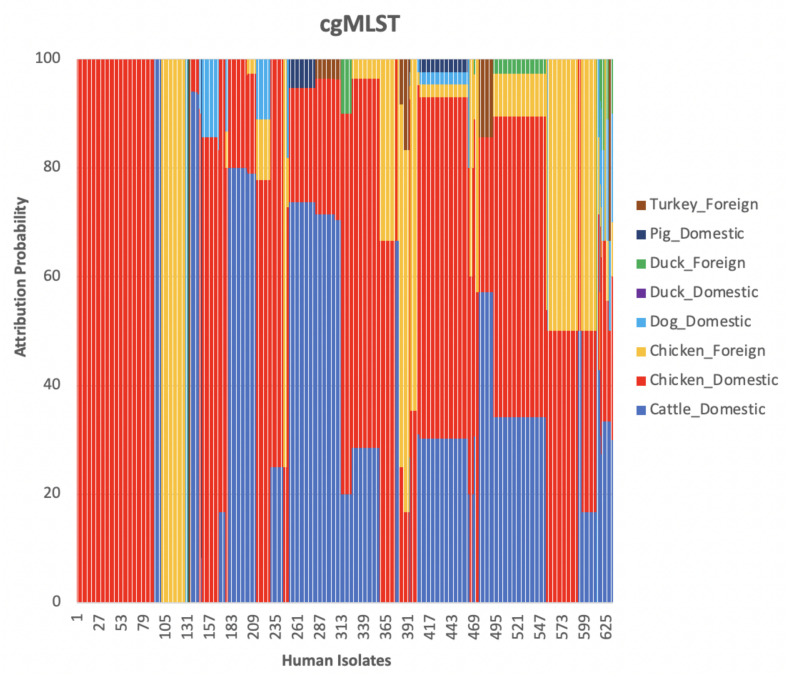
The probability of a human isolate to originate from each source as determined by source attribution analysis using the network-based approach on the cgMLST pairwise distance matrix.

**Figure 11:**
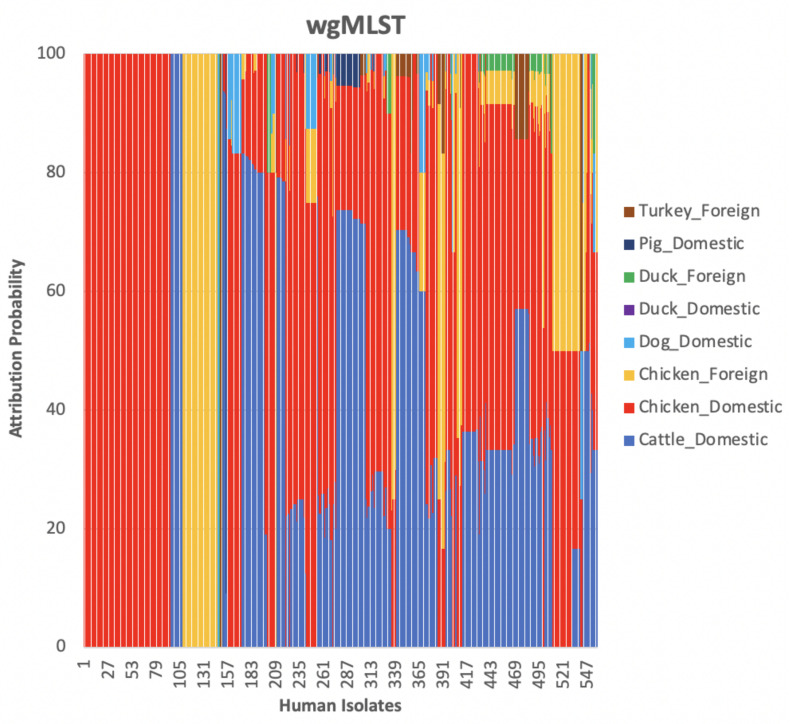
The probability of a human isolate to originate from each source as determined by source attribution analysis using the network-based approach on the wgMLST pairwise distance matrix.

**Figure 12:**
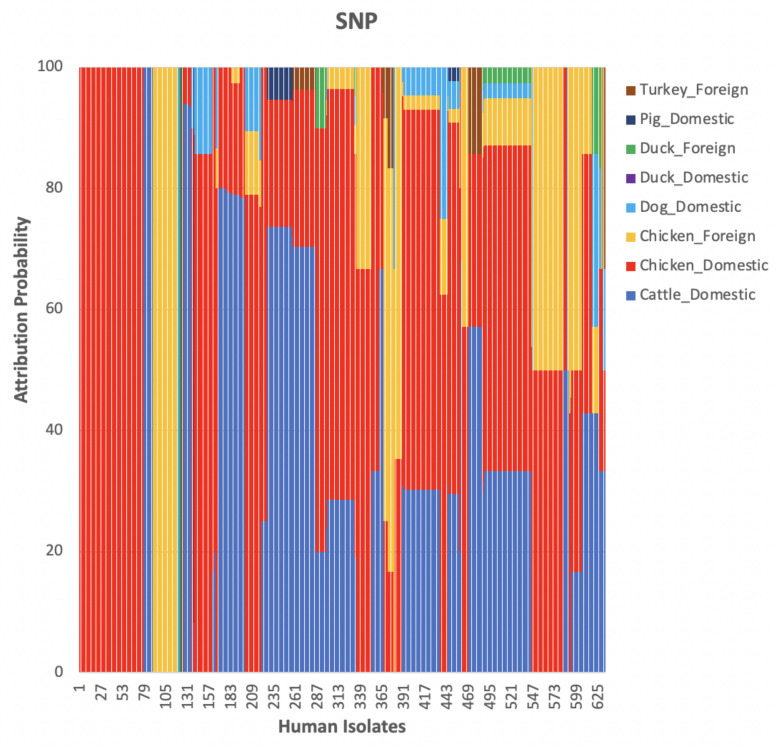
The probability of a human isolate to originate from each source as determined by source attribution analysis using the network-based approach on the SNP pairwise distance matrix.

The weighted network-based approach showed high specificity due to the number of links between each human sample and each animal source in all three networks (cgMLST, wgMLST and SNP), as observed in Figure 10, Figure 11 and Figure 12. We also observed 100% attribution of some human samples to less abundant sources, such as dogs from Denmark (Figure 10, Figure 11 and Figure 12), an indication that the algorithm used was not influenced by the sample size. Results from the network analysis comparing the three distance matrices as inputs suggested that the model is robust to the changes in the form of WGS used as model input (Figure 4, Figure 8, Figure 9 and Table 4, Table 5 and Table 6). In addition, since there was a class imbalance between isolates from Denmark and imported isolates, the finding that country of origin influenced cluster formation in this analysis should be further investigated using isolates from different countries or regions.

## 4. Conclusions

This study aimed at attributing human *Campylobacter* cases to different animal sources using a weighted network-based approach to exploit the potential of WGS data in conducting higher-resolution source attribution. We demonstrated that despite the high intra-species genetic diversity in *Campylobacter* [41], which would result in low discriminatory power in differentiating the different sources [4], the network analysis approach showed good discriminatory power, maximized cluster coherence and reduced the number of human isolates not attributed. The results obtained were robust to the different subtyping data used. However, the wgMLST distance matrix as input data may provide more accurate inputs than cgMLST and SNP, although this results in more human isolates not attributed to any sources. Chickens were the main cause of human *Campylobacter* infections. The analysis based on the country of origin of animal sources indicated that regionality affects cluster formation. Further studies are therefore recommended using data sets from different countries and different potential sources to confirm the reliability of the network-based approach as an alternative for source attribution.

## Figures and Tables

**Table 1:** Best threshold based on the animal of origin for networks based on SNP, cgMLST, and wgMLST distance matrices.

Performance	cgMLST	wgMLST	SNP
Best threshold	0.1141	0.0105	1715

**Table 2:** Coherent source clustering (CSC) according to country of origin, type of *campylobacter* and year of origin for networks based on SNP, cgMLST and wgMLST distance matrices.

CSC	cgMLST	wgMLST	SNP
Species	78%	79%	69%
Year	61%	64%	67%

**Table 3:** Number of attributed and not attributed human isolates.

Human Isolates	cgMLST	wgMLST	SNP
Attributed	632	558	633
Not attributed	85	159	86

**Table 4:** Confusion matrix obtained from source clustering results for cgMLST distance matrix.

True / Pred	Cattle.dk	Chkn.dk	Chkn.for	Dog.dk	Duck.dk	Duck.for	Pig.dk	Turkey.for
Cattle.dk	151	32	1	2	0	1	1	4
Chkn.dk	43	259	30	13	0	4	2	0
Chkn.for	2	20	87	0	0	1	0	2
Dog.dk	0	0	0	5	0	0	0	0
Duck.dk	0	0	0	0	7	0	0	0
Duck.for	0	0	0	0	0	17	0	0
Pig.dk	0	0	0	0	0	0	29	0
Turkey.for	0	0	0	0	0	0	0	4

True—true isolates of the same source; Pred - predicted isolates; Cattle.dk—cattle from Denmark; Chkn.dk—chickens from Denmark; Chkn.for—chickens from foreign countries; Dog.dk—dogs from Denmark; Duck.dk—ducks from Denmark; Duck.for—ducks from foreign countries; Pig.dk—pigs from Denmark; Turkey.for—turkeys from foreign countries.

**Table 5:** Confusion matrix obtained from source clustering results for wgMLST distance matrix.

True / Pred	Cattle.dk	Chkn.dk	Chkn.for	Dog.dk	Duck.dk	Duck.for	Pig.dk	Turkey.for
Cattle.dk	157	31	2	2	0	1	1	2
Chkn.dk	37	263	24	9	0	2	1	0
Chkn.for	2	14	92	0	0	2	0	2
Dog.dk	0	0	0	9	0	0	0	0
Duck.dk	0	0	0	0	7	0	0	0
Duck.for	0	0	0	0	0	19	0	0
Pig.dk	1	0	0	0	0	0	28	0
Turkey.for	2	1	0	1	0	0	0	5

True—true isolates of the same source; Pred—predicted isolates; Cattle.dk—cattle from Denmark; Chkn.dk—chickens from Denmark; Chkn.for—chickens from foreign countries; Dog.dk—dogs from Denmark; Duck.dk—ducks from Denmark; Duck.for—ducks from foreign countries; Pig.dk—pigs from Denmark; Turkey.for—turkeys from foreign countries.

**Table 6:** Confusion matrix obtained from source clustering results for SNP distance matrix.

True / Pred	Cattle.dk	Chkn.dk	Chkn.for	Dog.dk	Duck.dk	Duck.for	Pig.dk	Turkey.for
Cattle.dk	158	36	4	4	0	2	1	4
Chkn.dk	39	253	27	13	0	3	1	0
Chkn.for	2	20	89	0	0	1	0	2
Dog.dk	0	0	0	5	0	0	0	0
Duck.dk	0	0	0	0	7	0	0	0
Duck.for	0	0	0	0	0	18	0	0
Pig.dk	1	0	0	0	0	0	28	0
Turkey.for	0	0	0	0	0	0	0	3

True—true isolates of the same source; Pred—predicted isolates; Cattle.dk—cattle from Denmark; Chkn.dk—chickens from Denmark; Chkn.for—chickens from foreign countries; Dog.dk—dogs from Denmark; Duck.dk—ducks from Denmark; Duck.for—ducks from foreign countries; Pig.dk—pigs from Denmark; Turkey.for—turkeys from foreign countries.

## Data Availability

The data used can be found under the bioproject number set up by Statens Serum Institute PRJEB31119 [27].
